# Sleep Spindle Characteristics in Obstructive Sleep Apnea Syndrome (OSAS)

**DOI:** 10.3389/fneur.2021.598632

**Published:** 2021-02-25

**Authors:** Hiwa Mohammadi, Ardalan Aarabi, Mohammad Rezaei, Habibolah Khazaie, Serge Brand

**Affiliations:** ^1^Sleep Disorders Research Center, Kermanshah University of Medical Sciences, Kermanshah, Iran; ^2^Department of Neurology, School of Medicine, Kermanshah University of Medical Sciences, Kermanshah, Iran; ^3^Clinical Research Development Center, Imam Reza Hospital, Kermanshah University of Medical Sciences, Kermanshah, Iran; ^4^Laboratory of Functional Neuroscience and Pathologies (LNFP, EA4559), University Research Center (CURS), University Hospital of Amiens, Amiens, France; ^5^Faculty of Medicine, University of Picardie Jules Verne, Amiens, France; ^6^Department of Medical Physics and Biomedical Engineering, School of Medicine, Tehran University of Medical Sciences, Tehran, Iran; ^7^University of Basel, Psychiatric Clinics (UPK), Center for Affective, Stress and Sleep Disorders (ZASS), Basel, Switzerland; ^8^Department of Sport, Exercise and Health, Division of Sport Science and Psychosocial Health, University of Basel, Basel, Switzerland; ^9^Substance Abuse Prevention Research Center, Health Institute, Kermanshah University of Medical Sciences, Kermanshah, Iran; ^10^School of Medicine, Tehran University of Medical Sciences, Tehran, Iran

**Keywords:** spindle density, spindle duration, obstructive sleep apnea syndrome, N2, N3 apnea/hypopnea index

## Abstract

**Background:** We compared the density and duration of sleep spindles topographically in stage 2 and 3 of non-rapid eye movement sleep (N2 and N3) among adults diagnosed with Obstructive Sleep Apnea Syndrome (OSAS) and healthy controls.

**Materials and Methods:** Thirty-one individuals with OSAS (mean age: 48.50 years) and 23 healthy controls took part in the study. All participants underwent a whole night polysomnography. Additionally, those with OSAS were divided into mild, moderate and severe cases of OSAS.

**Results:** For N2, sleep spindle density did not significantly differ between participants with and without OSAS, or among those with mild, moderate and severe OSAS. For N3, *post-hoc* analyses revealed significantly higher spindle densities in healthy controls and individuals with mild OSAS than in those with moderate or severe OSAS. Last, in N2 a higher AHI was associated with a shorter sleep spindle duration.

**Conclusion:** OSAS is associated with a significantly lower spindle density in N3 and a shorter spindle duration in N2. Our results also revealed that, in contrast to moderate and severe OSAS, the sleep spindle characteristics of individuals with mild OSAS were very similar to those of healthy controls.

## Introduction

Obstructive sleep apnea syndrome (OSAS) is one of the most common breathing-related sleep disorders. The American Academy of Sleep Medicine (1) defines OSAS as repetitive episodes of complete or partial upper airway obstruction during sleep. Such complete or partial upper airway obstructions lead to a reduced blood oxygen saturation with brief arousals. Further, OSAS is diagnosed based on the patient complaints of daytime sleepiness, non-restorative sleep, fatigue or insomnia symptoms, along with awakening of sleep with breath holding, gasping or choking, and with their report of habitual snoring and/or breathing interruptions. Next, polysomnographic analyses (PSG) show five or more predominantly obstructive respiratory events [(obstructive and mixed apneas, hypopneas or respiratory effort-related arousals (RERAs)]. Relatedly, OSAS is diagnosed, if PSG analyses record 15 or more predominantly obstructive respiratory events (apneas, hypopneas, or RERAs) per hour (1).

As regards prevalence rates of OSAS, a meta-analysis reported the following figures: 3% of women, and 10% of men aged 30 to 49 years suffer from moderate to severe OSAS; 9% of women, and 17% of men aged 50 to 70 years suffer from moderate to severe OSA (2). Population-based studies reported prevalence rates of 23.4% for women and 49.7% for men (3, 4).

Obstructive sleep apnea syndrome is not only associated with a reduced sleep efficiency, but also with a lower quality of life (5) and an increased risk of systemic comorbidities such as cardiovascular diseases, hypertension, and metabolic syndrome (6–8). Relatedly, the most important epidemiological risk factors for OSAS are obesity and male gender (9, 10). In addition, higher OSAS is associated with a decreased cognitive executive performance and memory consolidation (11), with early changes in biomarkers for Alzheimer's Disease (12), and with the hypoactivation of brain regions associated with cognition (13, 14).

Within the broad range of encephalographic and sleep-related signals, sleep spindles are understood as 0.5 to 3 s episodes of 9–16 Hz electroencephalogram oscillations resulting from the intra-thalamic network of nucleus *Reticularis thalami* (nRt) and thalamocortical neurons during non-rapid eye movement (nREM) sleep (15–18). Sleep spindles are well-known as neurophysiological underpinnings of stage 2 nREM sleep (N2), but they can also occur in stage 3 nREM sleep (N3) (19).

Sleep spindles have attracted increased attention for their associations with cognitive, emotional and social processes: Sleep spindle activities are associated with neural structures and functions (20); it follows that sleep spindles are considered an important index both for typically developing neurocognitive processes and for sleep disorders (21, 22). To illustrate, a higher sleep spindle density was associated with increased memory consolidation (23), motor development, language abilities, learning, and cognitive performance (24). Among preschoolers, a higher spindle density predicted current and future cognitive-emotional and social skills (25–27). In contrast, a lower sleep spindle activity is observed among older individuals with dementia (28).

The pattern of sleep spindle indices among individuals with OSAS is inconsistent. As regards the assessment of sleep spindles, both visual and automatic spindle detection methods are used, along with spectral analysis techniques for detecting sigma band frequencies, either separately or in combination. In some studies individuals with OSAS had statistically similar patterns of spindle density and sigma power, compared to controls (29). In contrast, individuals with OSAS had a lower spindle density, slower spindle frequency, and lower sigma power, compared to healthy control (30–34).

As regards sleep stages, studies reported significant differences in sleep spindle characteristics between individuals with and without OSAS during all NREM stages but not during REM (33, 35, 36). Himanen et al. (37) observed an increased spindle frequency at the end of the night among healthy controls, but not among individuals with OSAS. Some studies reported significant differences in spindle characteristics between the two groups in REM sleep (38).

In children and adolescents, a lower spindle density in N2 but not in N3 was observed in children with mild OSAS, when compared to healthy controls (39). In contrast, Madaeva et al. (40) used a automatized software for sleep spindle detection and reported higher levels of spindles and spindle density, but a lower spindle amplitude and frequency during N2 in overweight adolescents with OSAS, compared to overweight or normal weight adolescents with no OSAS. No differences were observed for spindle duration between overweight adolescents with and without OSAS, and normal weight adolescents (40).

Last, spindle activity increases when OSAS is treated with positive airway pressure therapy (41–43). This effect could be understood as a normalization of sleep continuity, sleep architecture and sleep spindle activity in individuals with OSAS and treated with this therapy.

The pattern of mixed and inconclusive results noted above may reflect variations in sample sizes, age ranges, and different clinical criteria for recruiting and assessing participants, together with different methodologies of spindle detection at different sleep stages, and different outcome variables such as sigma band power, total number of spindles, spindle density and spindle duration.

Sleep spindles protect sleep from arousal-induced incidents, that is to say: Thalamocortical neuronal activation during spindle oscillations filters external sensory inputs to the neocortex and increases the threshold for response to external stimuli (15). Conversely, OSAS is likely to produce arousal as an internal arousal inducer, to disrupt sleep continuity and to lead to fragmented sleep. Given that both OSAS and lower sleep spindle activity were associated with a reduced memory consolidation (11, 23, 24), it is possible that the degradation of sleep spindles activity is responsible for the negative effects of OSAS on memory and cognition.

To summarize, the findings reported above do not allow to draw a uniform and coherent frame of association between OSAS and sleep spindle activity. Given this background, the aim of the present study was to shedding some further light on the associations between OSAS and sleep spindles. To this end, we analyzed the sleep spindle activity among individuals with mild, moderate and severe OSAS, and among healthy controls. We hypothesized that the spindle density and duration would differ between individuals with mild, moderate and severe OSAS, and compared to controls. Specifically, we expected that with higher OSAS sleep spindle patterns (spindle density and spindle duration) would be more impaired. The present study expands upon most previous work in that we also assessed individuals with severe OSAS. Further, there have been few studies of spindle duration, and the present study offers an opportunity to clarify the role of the spindle duration and its interaction with spindle density during both N2 and N3 stages. The present results could have clinical importance because individuals with OSAS are at increased risk of neurocognitive impairments and this may be at least partially explained by a deterioration in sleep spindle activity. The results from this study could also be important for practical reasons: Treating OSAS may also improve sleep spindle activities, resulting in improved behavior and cognitive performance.

## Materials and Methods

### Sample and Procedure

Individuals with OSAS and age-, sex-, and BMI-matched controls were invited to participate at the present study. Participants were fully informed about the aims of the study and the confidential data handling. Thereafter, they all signed the written informed consent. All participants underwent a thorough medical examination, and their sleep was objectively assessed via polysomnography. Sleep assessments took place at the Sleep Disorders Research Center (SDRC) of Kermanshah University of Medical Sciences (KUMS) between 2013 and 2016. The ethical committee of the Kermanshah University of Medical Sciences (KUMS, Kermanshah, Iran; code: KUMS.REC.1395.337) approved the study, which was performed in accordance with the ethical principles laid down in the seventh and current edition (44) of the Declaration of Helsinki.

### Samples

#### Individuals With Obstructive Sleep Apnea Syndrome (OSAS)

A total of 31 individuals with OSAS were enrolled in the study (mean age: 49.41 years, SD = 8.96; age range: 33–59 years; 84% males). Inclusion criteria were: (1) Age between 18 and 65 years; (2) Breathing-related sleep complaints, as assessed via a thorough clinical interview and based on polysomnographic data; (3) Compliance with the study conditions such as undergoing overnight polysomnography at the sleep research center; (4) Signed written informed consent. Exclusion criteria were: (1) Current severe psychiatric issues such as acute suicidality, acute psychosis, or severe substance use disorder (opium, alcohol, cannabis, amphetamines, methamphetamines; medications); (2) Severe chronic neurological or cardiovascular issues; (3) Unwilling or unable to comply with the study conditions. (4) Unable or unwilling to withdraw from sleep-altering medications (narcotics, antihistamines, etc.) 2 weeks before the polysomnographic assessment; (5) Periodic limb movements of sleep; (6) Shift work; (7) Female participants: currently pregnant or breastfeeding.

#### Healthy Controls

A total of 23 individuals without any kind of sleep disturbances were enrolled in the study (mean age: 45.17 years, SD = 10.73; age range: 34–56 years; 70% males). Inclusion criteria were: (1) Age between 18 and 65 years; (2) No sleep complaints, and above all no breathing-related sleep complaints, as assessed via a thorough clinical interview and based on polysomnographic data; (3) Compliance with the study conditions such as undergoing overnight polysomnography at the sleep research center; (4) Signed written informed consent. Exclusion criteria were identical to the exclusion criteria of individuals with OSAS.

### Detailed Data Collection

Participants were invited to the SDRC sleep laboratory (Kermanshah, Iran). They were advised not to have any coffee, tea, a heavy diet or a cigarette and not to snooze or sleep during the day. They were asked to arrive at the laboratory at 9 p.m. Then, they completed a short demographic questionnaire including age and sex, employee conditions, smoking, shift work; thereafter, their height and weight were measured by an experienced technician. Next, the PSG procedure was explained. The PSG room was standardized for any noise and visual stimulus based on international standards (1). An overnight PSG (SOMNOscreen plus®, Somnomedics, Randersacker, Germany) was performed for each participant. PSG recordings were started based on the individual's usual sleep habits, and each patient was recorded for a minimum of 7 h.

### Polysomnography

The PSG recording and scoring of sleep stages and respiratory events were performed by a sleep physician employing the American Academy of Sleep Medicine (AASM) guidelines (19). Measurement of PSG was based on the AASM guidelines according to the standard techniques, with monitoring of the electroencephalogram using frontal, central and occipital leads referenced to the mastoids (C3M2, F3M2, O1M2, C4M1, F4M1, and O2M1 derivations) according to the 10–20 system, electro-oculogram (EOG), electromyogram (EMG), flow (by oronasal thermistor and nasal air pressure transducer), thoracic and abdominal respiratory effort (induction plethysmography), oximetry, and body position. Respiration was monitored with oronasal thermocouples and nasal pressure transducers. Thoracoabdominal movements were monitored using piezoelectric strain gauges. Continuous pulse oximetry was also monitored.

The AASM criteria (1, 45) were applied to calculate the apnea/hypopnea index (AHI). Hypopnea is defined as a partial cessation of breathing; peak signal excursions drop by ≥ 30%, compared to the pre-event baseline for at least 10 s; further, compared to the pre-event baseline, oxygen saturation decreases by ≥ 3%; or the partial cessation of breathing is associated with an arousal. Apnea is defined as complete cessation of breathing; compared to the pre-event baseline the peak signal excursion drops by ≥ 90% for at least 10 s. AHI is calculated as the sum of apnea and hypopnea events per hour of sleep. AHI was used to confirm the clinical diagnosis. Further, based on the global AHI index, participants were classified into non-OSA (AHI <5), mild (5≤AHI<15), moderate (15≤AHI<30), and severe OSA (30≤AHI) groups. Wake index was calculated as the number of awakenings per hour of sleep. Arousal index is considered as the number of notable EEG shift toward a higher frequency for at least 3 s but no more than 15 s during all non-REM (NREM) stages per hour of sleep. Sleep efficiency (SE) was calculated as a ratio of total sleep time (TST) to time in bed × 100.

### EEG Analysis

All PSG data were digitized with a sampling rate of 256 Hz and the resolution of 16 bits, and then converted into the European Data Format (EDF) for further analysis in Matlab. The EEG signals were first band-pass filtered between 0.5 and 35 Hz using a 12th order Chebyshev Type II filter. EMG, ECG, and EOG artifacts were removed from the EEG signals by the independent component analysis (ICA). Thirty minutes of the EEG signals, 15 min from the beginning and 15 min from the end of each whole-night sleep recording were excluded from analysis to avoid unreliable data collected during wakefulness.

### Spindle Detection

We used the method proposed by Ferrarelli et al. (46) to detect sleep spindles through bandpass filtering and amplitude thresholding as implemented in Pareto-optimization software (46, 47). With few adjustable parameters, this method provides a good trade-off between performance and computational complexity (47). To differentiate spindles from EEG background activities and artifacts, the algorithm first preprocesses the EEG signal with a filter using the bandpass setting published by Warby et al. (48). The envelope of the rectified filtered signal peaks is then thresholded (p1 and p2) by two lower and upper amplitude thresholds used to identify spindle candidates. In this approach, to consider variations in signal amplitude between channels, thresholds are set relative to mean signal amplitude. A spindle is detected if its peak amplitude exceeds the upper threshold. The beginning and end of the spindle are determined before and after the spindle peak if the amplitude of the time series drops below the lower threshold. Finally, each spindle candidate is examined by the lower and upper duration criteria p3 and p4 sec. In our analysis, we first bandpass filtered the EEG data between 12 and 15 Hz (−3 dB at 12 and 15 Hz). We set the spindle lower (p1) and upper (p2) boundary threshold ratios to two and four times the average amplitude of the EEG signal for each channel, respectively, so as to achieve maximum sensitivity and low false detection rates. We then selected spindle candidates having a duration within [p3 p4], which were set to 0.5 to 3 s, respectively, as recommended in Liu et al. (47). For each channel, we then computed the spindle density (average number of spindles per minute) and average spindle duration (in sec) for N2 and N3. Spindle density in N2 and N3 was calculated as the ratio of the number of sleep spindles to the number of minutes of N2 and N3 separately.

### Statistical Analysis

Comparison of sociodemographic (age, sex) and anthropometric dimensions (BMI) between participants without and with mild, moderate, and severe OSAS was made via a series of *X*^2^-tests and ANOVAs. Sleep characteristics and spindle parameters were compared between the four groups via ANOVAs. Eta squared was used to measure effect size. *Post-hoc* Tukey test were performed for multiple comparisons between the four groups. The statistical comparisons were made on a channel-by-channel basis. Pearson's correlations were computed for associations between AHI, spindle density and spindle duration for all electrode sites (F3, F4, C3, C4, O1, O2). All statistical computations were performed with SPSS® 25.0 (IBM Corporation, Armonk NY, USA) for Windows®.

## Results

### Sociodemographic and Anthropometric Findings

Table 1 provides the descriptive and inferential statistical indices for age, sex, and BMI, between the four groups. There were no significant differences between groups. Accordingly, age, sex, and BMI were not introduced as possible confounders.

**Table 1:** Sociodemographic and anthropometric descriptive and inferential statistical indices of healthy controls and individuals with mild, moderate and severe Obstructive Sleep Apnea.

	**Groups**				**Statistics**
	**Healthy controls**	**Mild OSAs**	**Moderate OSAs**	**Severe OSAs**	
*N*	23	8	8	15	
	n/n	n/n	n/n	n/n	
Sex (male/female)	16/7	5/3	6/2	13/2	*X*^2^ (*N* = 54, df = 3) = 2.04, *p* = 0.56
	M (SD)	M (SD)	M (SD)	M (SD)	
Age (years)	45.17 (10.73)	44.12 (10.75)	49.37 (8.86)	52.26 (7.11)	*F*_(3, 51)_ = 2.11, *p* = 0.11
BMI	27.96 (4.96)	28.66 (3.32)	31.25 (3.97)	31.12 (5.04)	*F*_(3, 51)_ = 1.89, *p* = 0.14

*BMI, Body Mass Index*.

### Sleep Dimensions

Table 2 provides the descriptive and inferential statistical indices for all sleep dimensions, including AHI and O_2_% saturation, between the four groups.

**Table 2:** Descriptive and inferential statistical indices of sleep-disordered breathing and objective sleep-EEG parameters of healthy controls and individuals with mild, moderate and severe Obstructive Sleep Apnea.

	**Groups**				**Statistics**
	**Healthy controls**	**Mild OSAs**	**Moderate OSAs**	**Severe OSAs**	
*N*	23	8	8	15	*F*_(3, 51)_; partial etM2; *post-hoc* tests
	M (SD)	M (SD)	M (SD)	M (SD)	
AHI	1.55 (0.97)	10.78 (1.63)	23.81 (3.33)	53.28 (15.96)	114.01**; 0.87 (M); HC < MiOSAs, MoOSAs, SeOSAs; MiOSAs < MoOSAs, SeOSAs; MoOSAs < SeOSAS
TST (h)	7.21 (0.29)	6.99 (0.53)	6.30 (0.88)	6.63 (0.94)	4.78**; 0.22 (L); HC = MiOSAs; HC > MoOSAs, SeOSAs; MiOSAs = MoOSAs = SeOSAs
SE	92.92 (3.72)	93.42 (6.67)	80.72 (11.60)	86.11 (7.92)	8.05**, 0.33 (L); HC = MiOSAs; HC > MoOSAs, SeOSAs; MiOSAs > MoOSAs; MiOSAs = SeOSAS; MoOSAs = SeOSAs
N1%	32.16 (13.90)	39.25 (14.01)	41.61 (23.29)	56.59 (17.79)	6.57**,.28 (L); HC < SeOSAs; MiOSAs = MoOSAs = SeOSAs
N2%	22.44 (13.66)	20.13 (13.38)	28.20 (24.01)	20.92 (11.62)	0.50,.03 (S); HC = MiOSAs = MoOSAs = SeOSAs
N3%	30.44 (15.42)	29.61 (19.37)	12.96 (11.63)	9.36 (10.24)	8.35**,.33 (L); HC > MoOSAs, SeOSAs; MiOSAs > SeOSAs; MoOSAs = SeOSAs
REM%	14.70 (15.73)	10.98 (10.92)	16.05 (15.86)	11.80 (10.63)	0.39, 0.02 (S); HC = MiOSAs = MoOSAs = SeOSAs
Arousals index	25.83 (5.29)	25.32 (3.85)	24.23 (4.76)	31.96 (11.20)	3.04*, 0.15 (L); HC = MiOSAs = MoOSAs = SeOSAs
Wake index	2.45 (1.35)	2.50 (3.13)	5.87 (5.22)	4.81 (3.14)	3.92**, 0.19 (L); HC < MoOSAs; MiOSAs = MoOSAs = SeOSAs
Minimum SpO2%	90.30 (1.76)	82.87 (6.46)	75.50 (11.09)	67.33 (12.48)	24.74***, 0.60 (L); HC > MoOSAs, SeOSAs; MiOSAs > SeOSAs; MoOSAS = SeOSAs

AHI, apnea/hypapnea index; TST, total sleep time (hour); SE, sleep efficiency; N1%, percent of N1 stage; N2%, percent of N2 stage; N3%, percent of N3 stage; REM%, percent of REM stage; OSAS, Obstructive Sleep Apnea Syndrome; *p < 0.05; **p < 0.01; ***p < 0.001; (S), small effect size; (M), medium effect size; (L), large effect size; >, longer than; <, shorter than, =, as long as; HC, healthy controls; MiOSAs, mild Obstructive Sleep Apnea Syndrome; MoOSAs, moderate Obstructive Sleep Apnea Syndrome; SeOSAs, severe Obstructive Sleep Apnea Syndrome.

A series of ANOVAs was performed to compare sleep characteristics between the groups. AHI, TST, SE, N1, and N3 sleep percent, sleep arousal index, wake index, and minimum sleep O_2_% saturation differed significantly between the groups. More specifically, SE and N3% was significantly lower in the groups with moderate and severe OSA, compared to the groups with no and mild OSA. N1% was significantly higher in the group with severe OSA, compared to the groups with no, mild and moderate OSA. N1% did not differ between the groups with no, mild and moderate OSA. No statistically significant group differences were observed for N2% and REM%. The arousal index was significantly higher in the group with severe OSA, compared to the groups with no, mild and moderate OSA. Wake index was statistically significantly higher in the groups of moderate and severe OSA, compared to the groups with no and mild OSA.

### Spindles Characteristics

**Figure 1:**
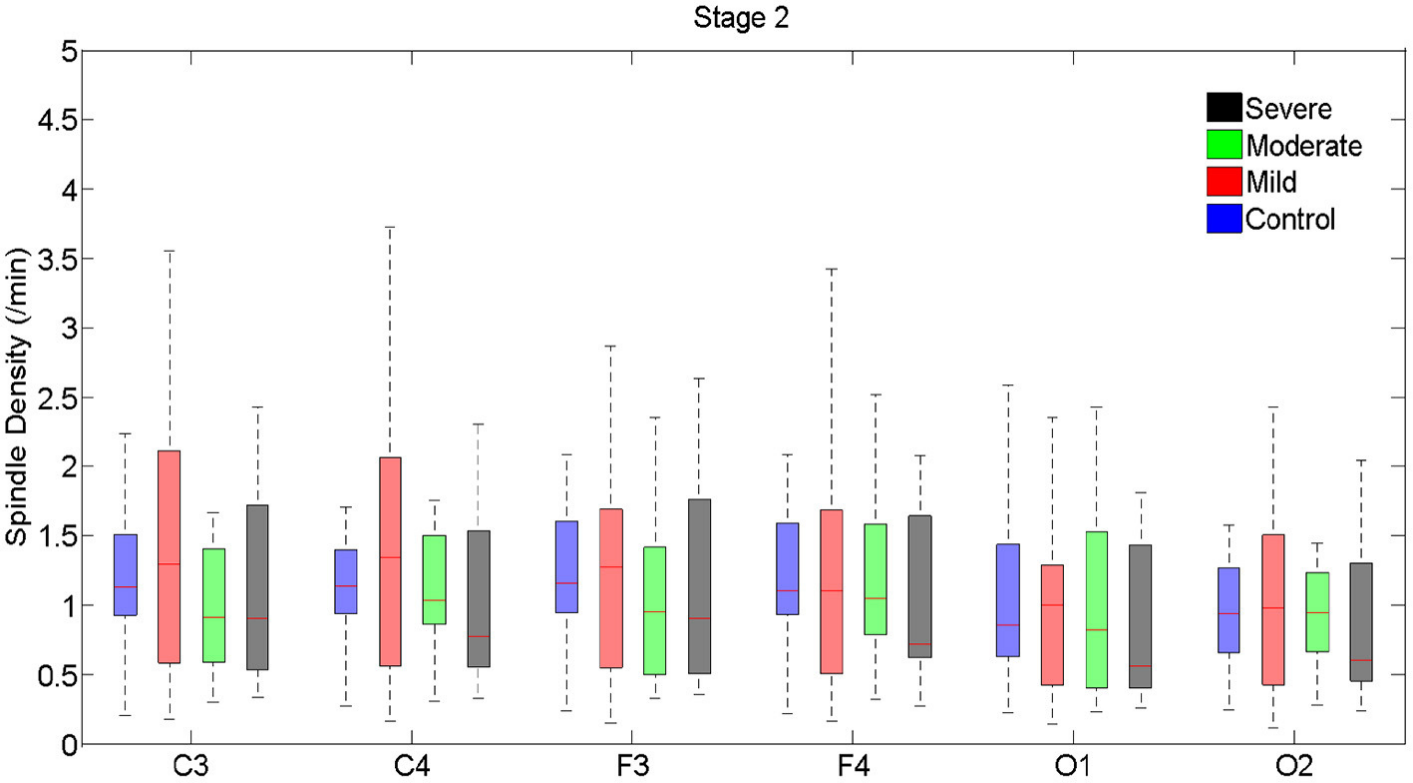
Boxplots displaying the median and interquartile range of sleep spindle density between patients with OSA and normal sleepers in N2 in different measurement sites.

**Figure 2:**
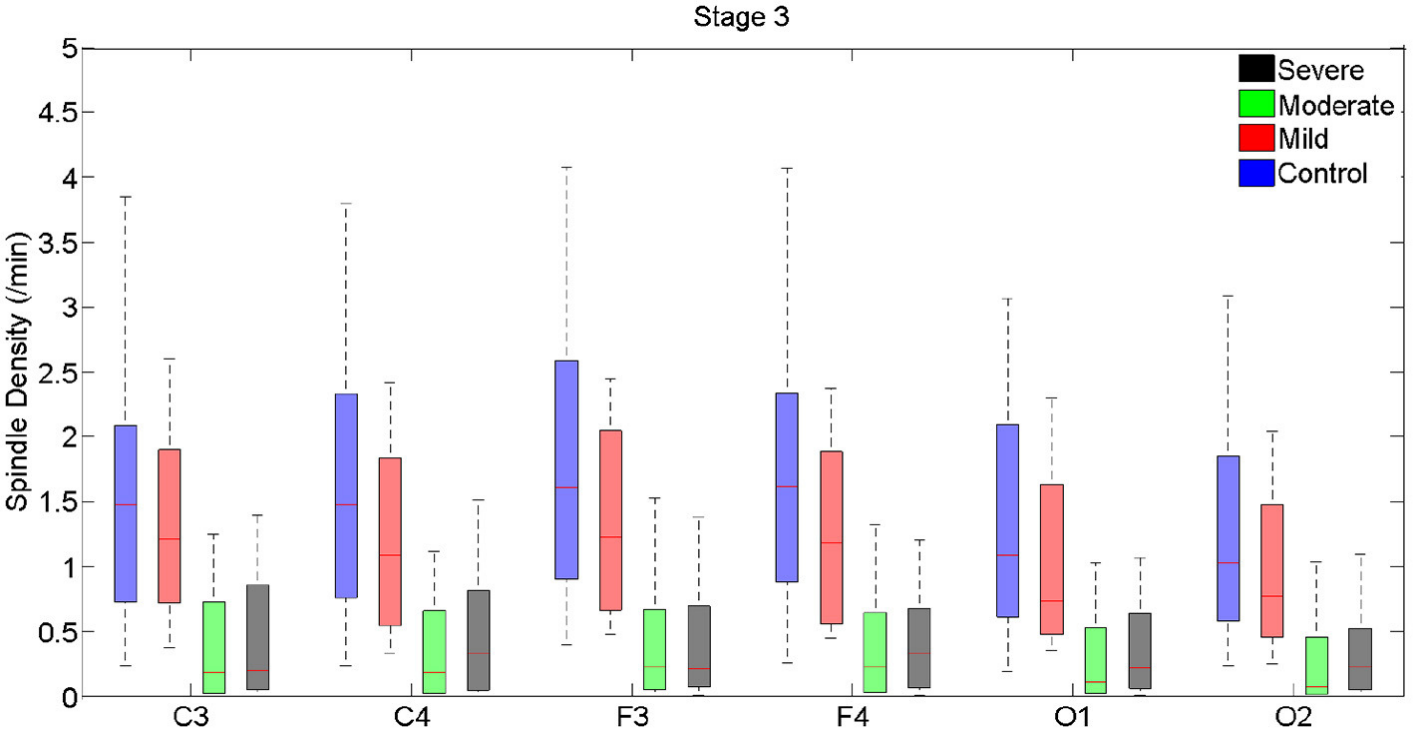
Boxplots displaying the median and interquartile range of sleep spindle density between patients with OSA and normal sleepers in N3 in different measurement sites.

**Figure 3:**
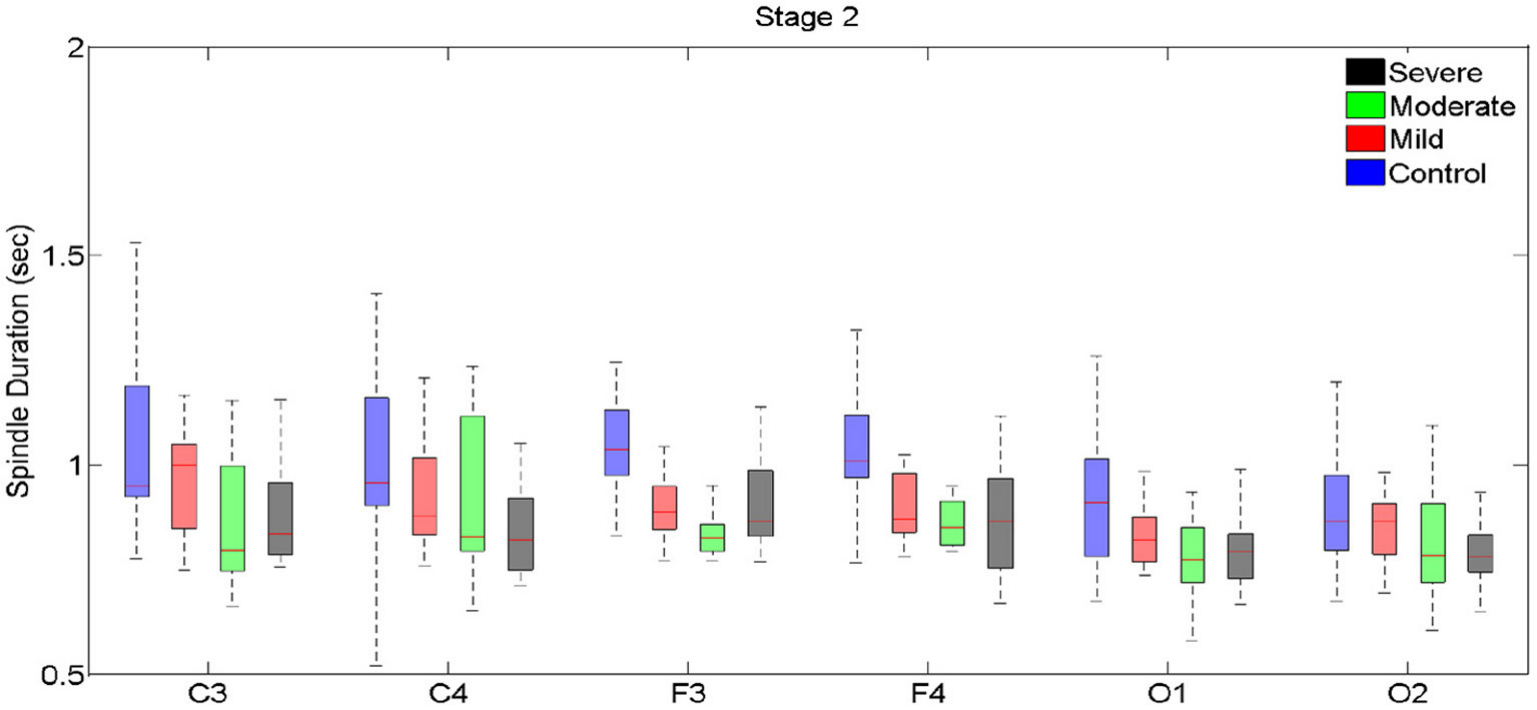
Boxplots displaying the median and interquartile range of sleep spindle duration for patients with OSA and normal sleepers in N2 in different measurement sites.

**Figure 4:**
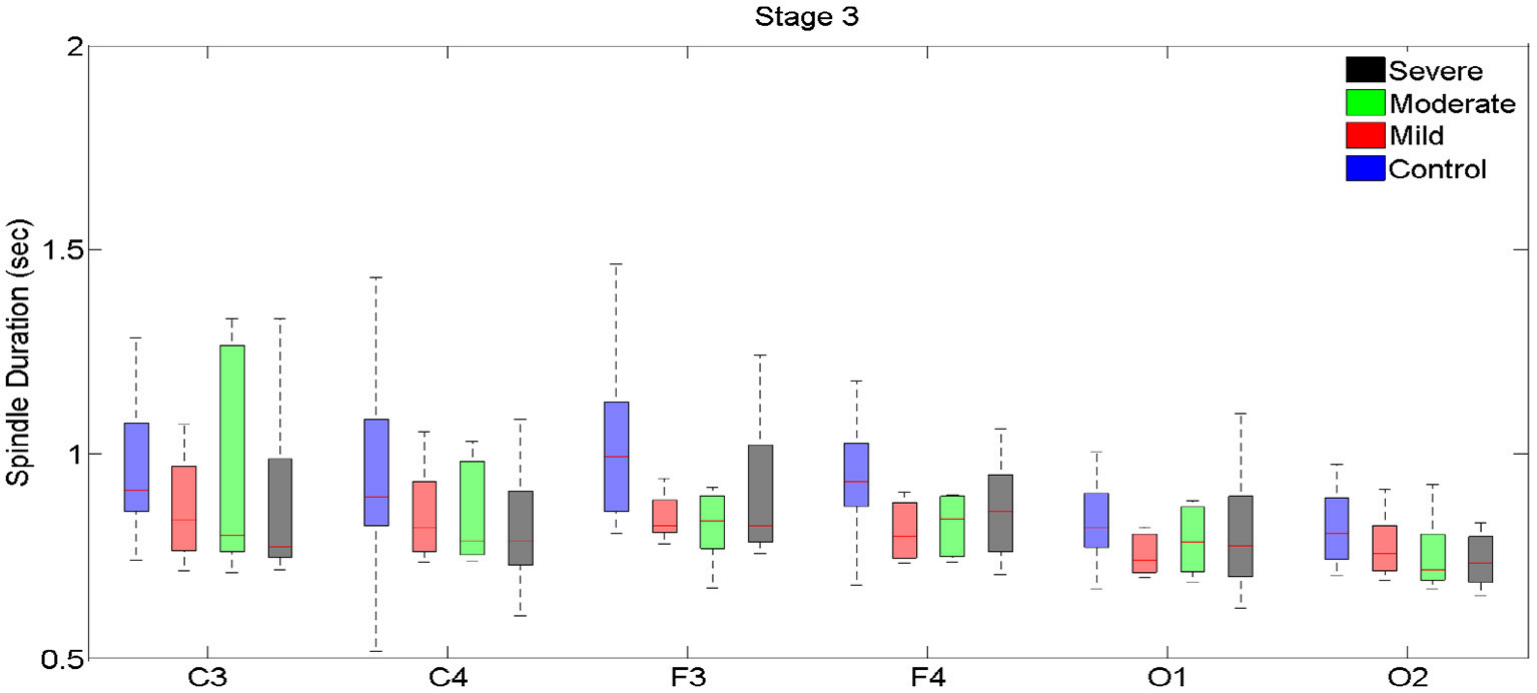
Boxplots displaying the median and interquartile range of sleep spindle duration for patients with OSA and normal sleepers in t N3 in different measurement sites.

Tables 3, 4 and Figures 1–4 (along with Supplementary Tables 1–4) provide the descriptive and inferential statistical indices of spindle-related characteristics between the four groups. Spindle density was compared between the three OSA groups and control separately for N2 and N3. While descriptively spindle density varied as a function of AHI in N2, for all electrode sites there were no statistically significant mean differences between groups (Table 3, Figure 1, Supplementary Table 1).

**Table 3:** Descriptive and inferential statistical indices of spindle density indices between healthy controls, and individuals with mild, moderate, and severe Obstructive Sleep Apnea.

	**Groups**				**Statistics**
	**Healthy controls**	**Mild OSAs**	**Moderate OSAs**	**Severe OSAs**	
*N*	23	8	8	15	
	M (SD)	M (SD)	M (SD)	M (SD)	*F*_(3, 51)_; partial etM2; *post-hoc* tests
**N2 stage**					
F3-M2	1.07 (0.15)	0.89 (0.08)	0.83 (0.07)	0.92 (0.14)	9.28**; 0.36 (L); HC > MiOSAs; MoOSAs; SeOSAs; MiOSAs = MoOSAs = SeOSAs;
F4-M1	1.06 (0.16)	0.90 (0.09)	0.86 (0.14)	0.88 (0.13)	7.09**; 0.30 (L); HC = MoOSAs = SeOSAs;
C3-M2	1.09 (0.26)	0.96 (0.14)	0.94 (0.36)	0.88 (0.12)	2.46^(^*^)^; 0.13 (M): HC = MoOSAs = SeOSAs;
C4-M1	1.03 (0.25)	0.93 (0.14)	0.92 (0.21)	0.84 (0.10)	2.86*; 0.15 (L); HC = MoOSAs = SeOSAs;
O1-M2	0.91 (0.14)	0.83 (0.08)	0.78 (0.11)	0.79 (0.84)	4.28**; 0.20 (L); HC = MoOSAs = SeOSAs;
O2-M1	0.90 (0.13)	0.85 (0.09)	0.82 (0.15)	0.81 (0.11)	1.94; 0.10 (M); HC = MoOSAs = SeOSAs;
**N3 stage**					
F3-M2	0.99 (0.17)	0.85 (0.06)	0.86 (0.17)	0.90 (0.16)	2.98*; 0.15 (L); HC > MiOSAs; MoOSAs; SeOSAs; MiOSAS > SeOSAs; MoOSAs = SeOSAs
F4-M1	0.97 (0.16)	0.81 (0.07)	0.87 (0.17)	0.89 (0.17)	2.43^(^*^)^; 0.13 (M); HC > MoOSAs; SeOSAs; MoOSAs = MiOSAS = SeOSAs
C3-M2	1.01 (0.25)	0.87 (0.13)	1.22 (0.96)	0.91 (0.21)	1.32; 0.07 (M); HC < MoOSAs; HC > SeOSAs; MiOSAS = SeOSAs
C4-M1	0.94 (0.20)	0.85 (0.12)	0.90 (0.25)	0.82 (0.13)	1.36; 0.08 (M); HC > MiOSAs; SeOSAs; MiOSAs = MoOSAs = SeOSAs
O1-M2	0.83 (0.09)	0.79 (0.14)	0.91 (0.37)	0.80 (0.13)	0.76; 0.04 (S); HC < MiOSAS; HC > SeOSAs; MiOSAs = MoOSAs = SeOSAs
O2-M1	0.81 (0.08)	0.78 (0.08)	0.76 (0.09)	0.76 (0.11)	1.26; 0.07 (M); HC > MoOSAs; SeOSAs; MiOSAs = MoOSAs = SeOSAs

OSAS, Obstructive Sleep Apnea Syndrome; (*)p < 0.1; *p < 0.05; **p < 0.01; (S), small effect size; (M), medium effect size; (L), large effect size; >, longer than; <, shorter than, =, as long as; HC, healthy controls; MiOSAs, mild Obstructive Sleep Apnea Syndrome; MoOSAs, moderate Obstructive Sleep Apnea Syndrome; SeOSAs, severe Obstructive Sleep Apnea Syndrome.

**Table 4:** Descriptive and inferential statistical indices of spindle duration between healthy controls, and individuals with mild, moderate and severe Obstructive Sleep Apnea.

	**Groups**				**Statistics**
	**Healthy controls**	**Mild OSAs**	**Moderate OSAs**	**Severe OSAs**	
*N*	23	8	8	15	
	M (SD)	M (SD)	M (SD)	M (SD)	*F*_(3, 51)_; partial etM2; *post-hoc* tests
**N2 stage**					
F3-M2	1.07 (0.15)	0.89 (0.08)	0.83 (0.07)	0.92 (0.14)	9.28**; 0.36 (L); HC > MiOSAS, MoOSAs, SeOSAs; MiOSAs = MoOSAs = SeOSAs
F4-M1	1.06 (0.16)	0.90 (0.09)	0.86 (0.14)	0.88 (0.13)	7.09**; 0.30 (L); HC > MiOSAS, MoOSAs, SeOSAs; MiOSAs = MoOSAs = SeOSAs
C3-M2	1.09 (0.26)	0.96 (0.14)	0.94 (0.36)	0.88 (0.12)	2.46^(^*^)^; 0.13 (M) HC = MiOSAs = MoOSAs = SeOSAs
C4-M1	1.03 (0.25)	0.93 (0.14)	0.92 (0.21)	0.84 (0.10)	2.86*; 0.15 (L); HC > SeOSAs; MiOSAs = MoOSAs = SeOSAs
O1-M2	0.91 (0.14)	0.83 (0.08)	0.78 (0.11)	0.79 (0.84)	4.28**; 0.20 (L): HC > MoOSAs, SeOSAs;
O2-M1	0.90 (0.13)	0.85 (0.09)	0.82 (0.15)	0.81 (0.11)	1.94; 0.10 (M); HC= SeOSAs; MiOSAs = MoOSAs = SeOSAs
**N3 stage**					
F3-M2	0.99 (0.17)	0.85 (0.06)	0.86 (0.17)	0.90 (0.16)	2.98*; 0.15 (L): HC= SeOSAs; MiOSAs = MoOSAs = SeOSAs
F4-M1	0.97 (0.16)	0.81 (0.07)	0.87 (0.17)	0.89 (0.17)	2.43^(^*^)^; 0.13 (M); HC= SeOSAs; MiOSAs = MoOSAs = SeOSAs
C3-M2	1.01 (0.25)	0.87 (0.13)	1.22 (0.96)	0.91 (0.21)	1.32; 0.07 (M); HC= SeOSAs; MiOSAs = MoOSAs = SeOSAs
C4-M1	0.94 (0.20)	0.85 (0.12)	0.90 (0.25)	0.82 (0.13)	1.36; 0.08 (M); HC= SeOSAs; MiOSAs = MoOSAs = SeOSAs
O1-M2	0.83 (0.09)	0.79 (0.14)	0.91 (0.37)	0.80 (0.13)	0.76; 0.04 (S); HC= SeOSAs; MiOSAs = MoOSAs = SeOSAs
O2-M1	0.81 (0.08)	0.78 (0.08)	0.76 (0.09)	0.76 (0.11)	1.26; 0.07 (M); HC= SeOSAs; MiOSAs = MoOSAs = SeOSAs

OSAS, Obstructive Sleep Apnea Syndrome; (*)p < 0.1; *p < 0.05; **p < 0.01; (S), small effect size; (M), medium effect size; (L), large effect size; >, longer than; <, shorter than, =, as long as; HC, healthy controls; MiOSAs, mild Obstructive Sleep Apnea Syndrome; MoOSAs, moderate Obstructive Sleep Apnea Syndrome; SeOSAs, severe Obstructive Sleep Apnea Syndrome.

In N3, spindle density progressively declined from the control group to the group with severe OSAS (Figure 2, Supplementary Table 2). Differences were statistically significant between control and the groups with moderate and severe OSAS; no statistically significant mean differences were observed between the groups with no and mild OSA. Further, no statistically significant mean differences were observed between the groups with mild, moderate and severe OSA (Table 3, Figure 2, Supplementary Table 2). These patterns of results were observed in all electrode sites; however, the difference was higher for frontal than for occipital sites.

Spindle duration was also compared between the groups of no, mild, moderate and severe OSA, and separately for N2 and N3.

Spindle duration differed significantly between the groups with no, mild, moderate and severe OSA in N2, but not in N3.

In N2, for all electrodes except for C3 and O2, there was a statistically significant mean difference in spindle duration between the four groups (Table 4, Figure 3, Supplementary Table 3). Specifically, for frontal electrodes, the Tukey *post-hoc* analysis showed statistically significant differences between the groups with no, mild, moderate and severe OSA. For central electrodes, compared to the group with no, mild and moderate OSA, the group with severe OSA had a significantly shorter spindle duration.

In N3, no significant differences in spindle duration were found between the groups with no, mild, moderate and severe OSA (Table 4, Figure 4, Supplementary Table 4).

### Correlations Between AHI and Spindle-Related Dimensions

**Figure 5:**
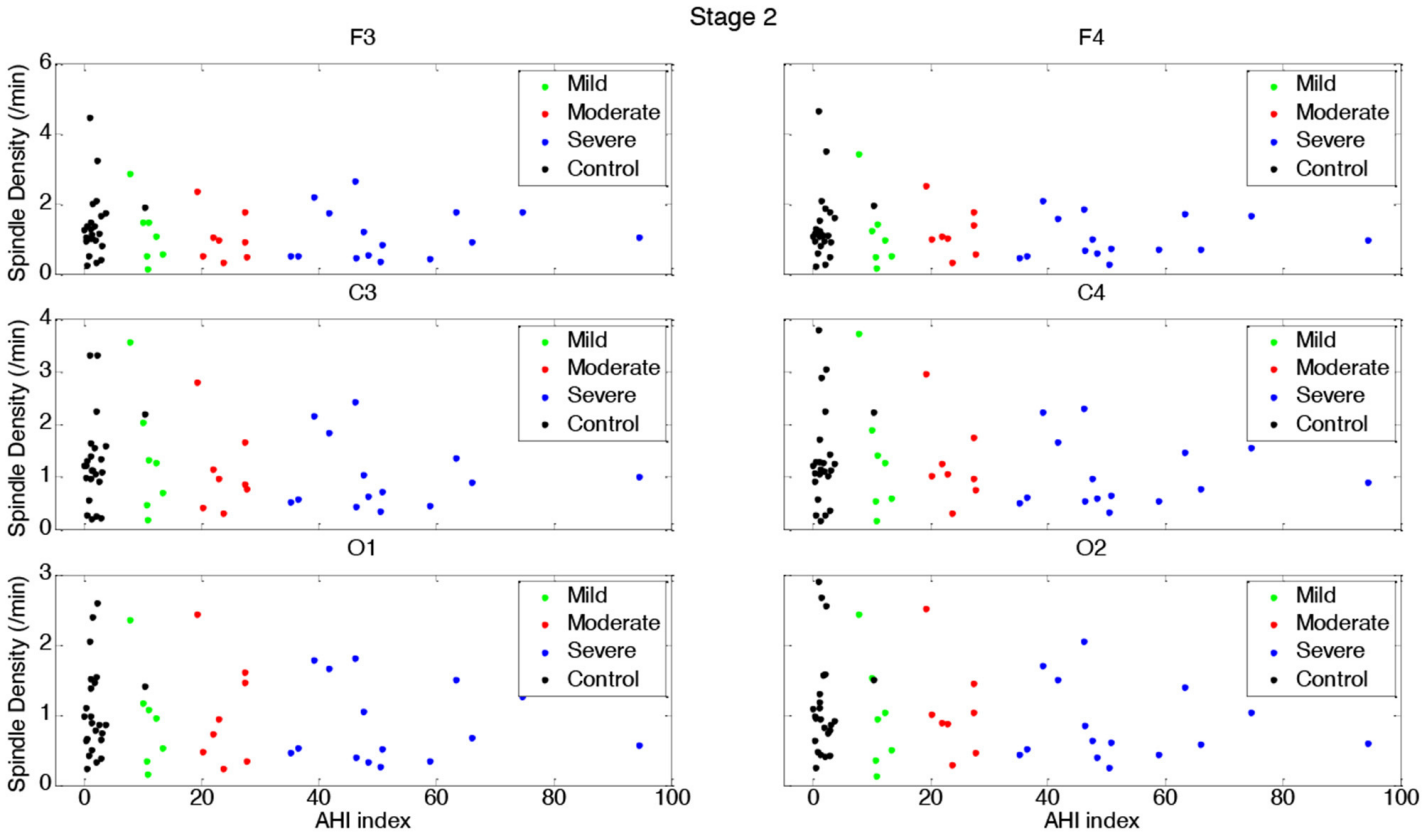
Spindle density distribution vs. AHI in N2 for each group.

**Figure 6:**
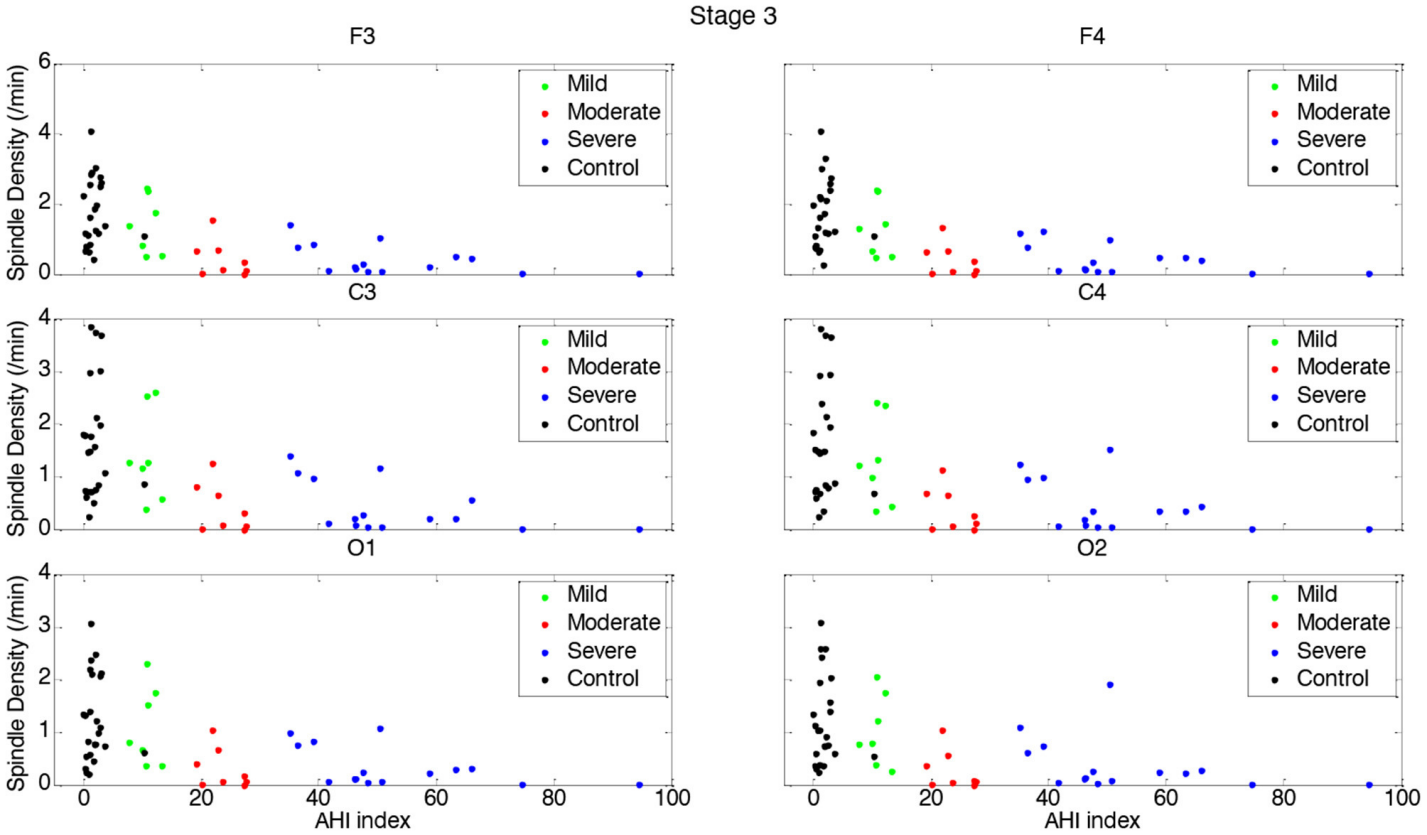
Spindle density distribution vs. AHI in N3 for each group.

**Figure 7:**
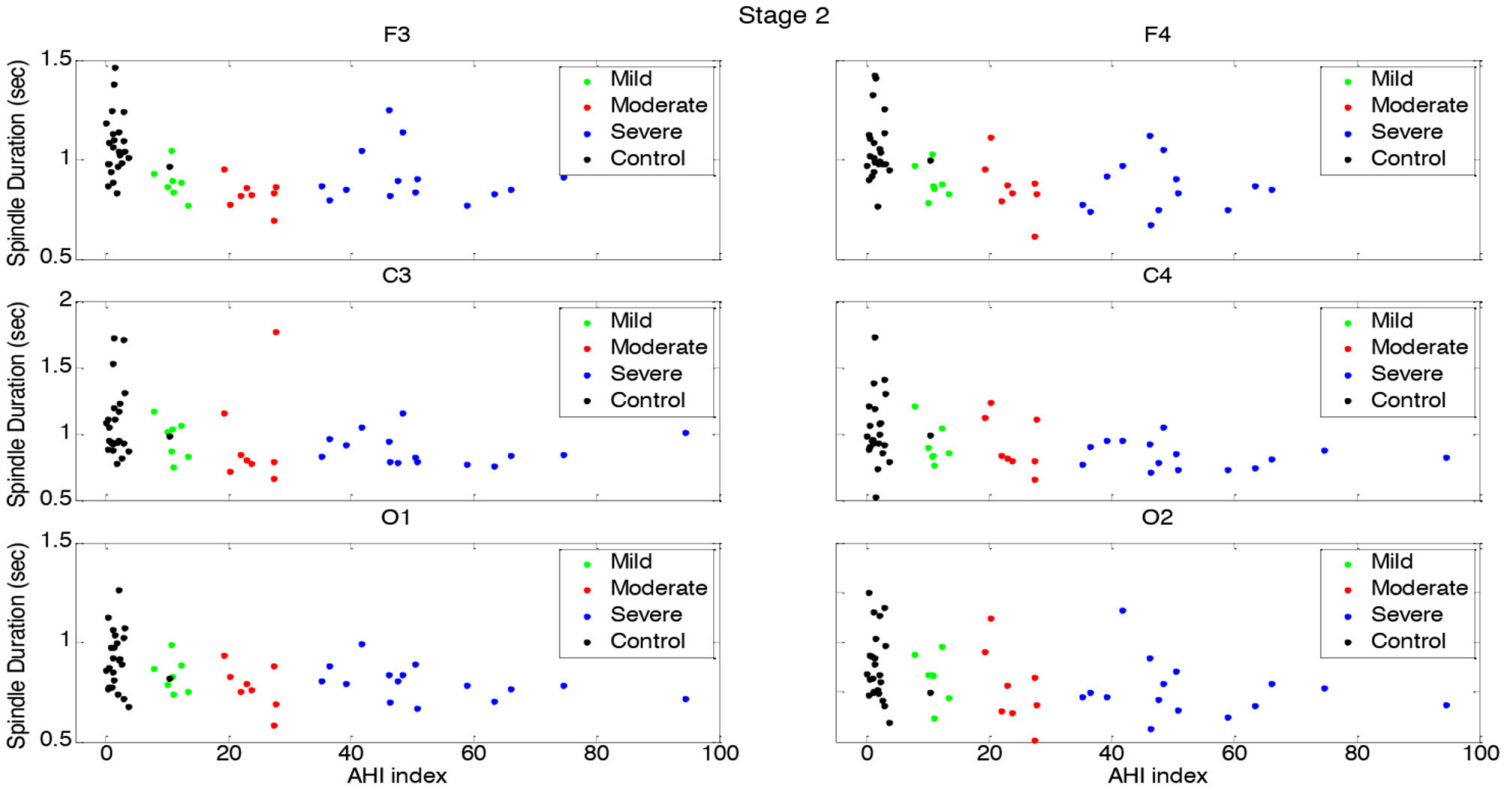
Spindle duration distribution vs. AHI in N2 for each group.

**Figure 8:**
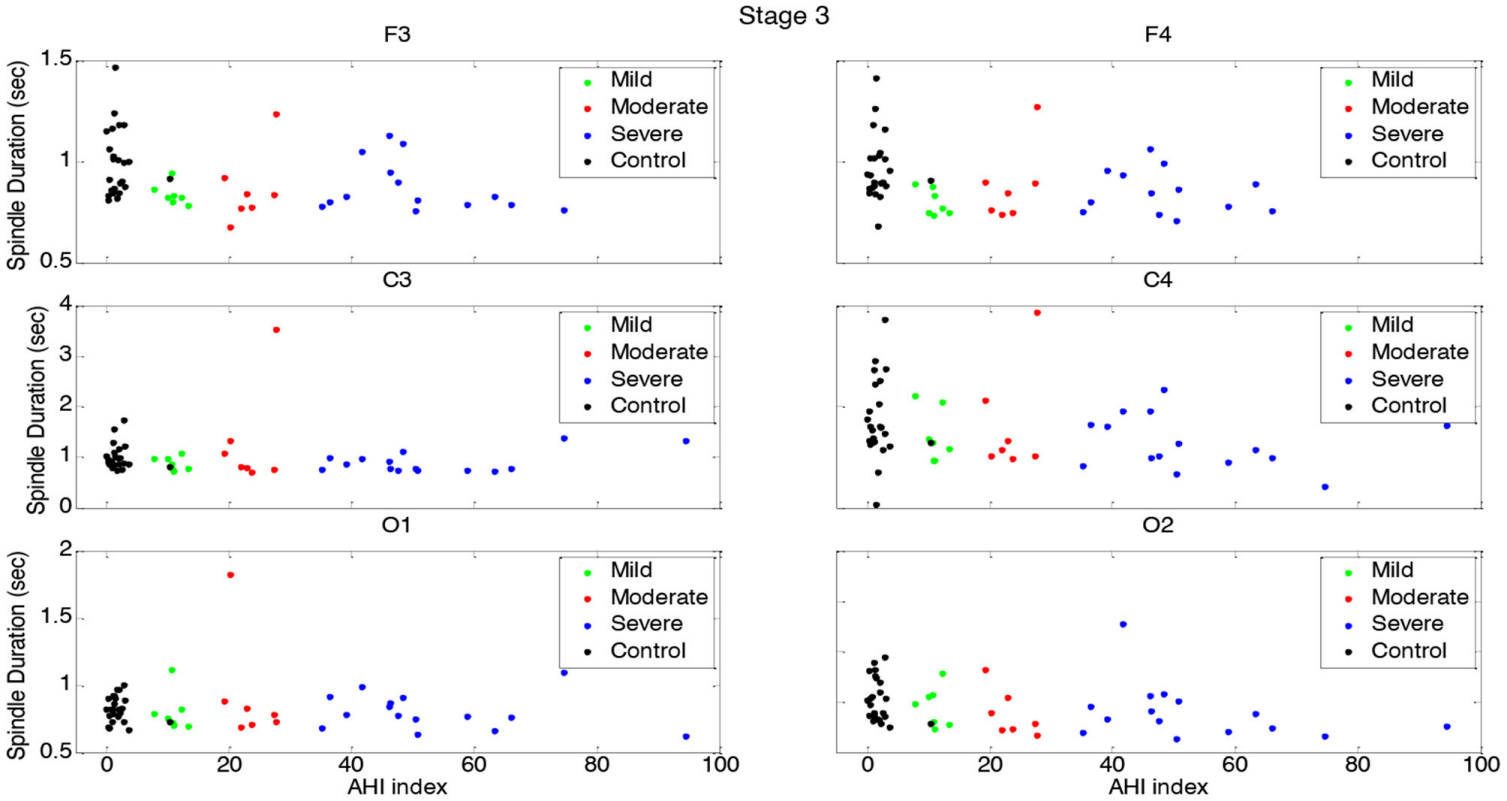
Spindle duration vs. AHI in N3 for each group.

Table 5, Figures 5–8 (Supplementary Material) provide the correlations between AHI and spindle-related dimensions, separately for the four groups. No significant correlations were found between AHI and spindle density in N2 (Table 5, Figure 5). Significant negative correlations were found between AHI and spindle density for all electrodes in N3 (*r* < −0.54; *p* < 0.01; Table 5, Figure 6).

**Table 5:** Correlation coefficients between apnea/hypopnea indices (AHI) and sleep spindle density and duration, separately for different electrodes for the whole sample.

	**Correlation coefficient**	***p***
**Density N2 stage**		
F3-A2	−0.11	0.40
F4-A1	−0.13	0.32
C3-A2	−0.11	0.42
C4-A1	−0.13	0.33
O1-A2	−0.12	0.37
O2-A1	−0.18	0.19
**Density N3 stage**		
F3-A2	−0.61	<0.01
F4-A1	−0.60	<0.01
C3-A2	−0.57	<0.01
C4-A1	−0.56	<0.01
O1-A2	−0.54	<0.01
O2-A1	−0.54	<0.01
**Duration N2 stage**		
F3-A2	−0.48	<0.01
F4-A1	−0.47	<0.01
C3-A2	−0.41	<0.01
C4-A1	−0.44	<0.01
O1-A2	−0.42	<0.01
O2-A1	−0.39	<0.01
**Duration N3 stage**		
F3-A2	−0.34	0.01
F4-A1	−0.23	0.08
C3-A2	−0.23	0.09
C4-A1	−0.30	0.02
O1-A2	−0.15	0.27
O2-A1	−0.38	<0.01

Figure 7 depicts the gradual decline in spindle duration as a function of AHI in N2. This result was replicated for all electrode sites. In N2, spindle duration was negatively correlated with AHI for all electrodes (*r* < −0.39; *p* < 0.01) (Table 5). Although no statistically significant differences were found in spindle duration between the groups for any electrode site (Table 4), a tendency to spindles with shorter durations was observed with increasing AHI in N3 and for all electrodes (Figure 8). The correlation between AHI and spindle duration in N3 was only significant for F3, C4, and O2 electrodes (*r* < −0.30; *p* < 0.02) (Table 5).

## Discussion

In the present study, sleep spindle density and sleep spindle duration, along with sleep architecture indices, were investigated topographically in N2 and N3 sleep-EEG signals in a sample of individuals with mild, moderate and severe OSAS and in healthy controls. The key findings were as follows: (1) with increasing OSA severity sleep spindle density decreased in N3, but not in N2. (2) no differences in spindle density were observed between individuals with no and mild OSA, and between individuals with moderate and severe OSA. (3) Such differences were found in the frontal, but not in the occipital area. (4) A higher OSA severity was associated with a shorter spindle duration in N2, but not in N3. (5) A higher spindle density was associated with a lower AHI index in N2, but not in N3. (6) Sleep architecture indices did not differ between individuals with no and mild OSA. (7) Sleep efficiency and total sleep time were significantly lower among participants with moderate and severe OSAS than in participants with mild OSAS or no OSAS. The present pattern of results adds to the current literature in an important way, in that we confirmed that in individuals with OSA sleep spindle density and duration vary in a sophisticated fashion, also depending from the sleep stage (N2 vs. N3), the topographical area (frontal area vs. occipital area) and from the AHI index.

The results on sleep architecture and sleep spindles are considered now in turn.

For sleep architecture, N2% and REM% did not significantly differ between participants without and with mild, moderate and severe OSAS. In contrast, N3% was significantly lower in participants with moderate and severe OSAS, compared to participants with no or mild OSAS; further, N1% was significantly higher in participants with severe OSA than in the other three groups. The pattern of results observed in the present study appears to contradict previous results. Swihart et al. (49) stated that OSA did not influence PSG dimensions such as total sleep time or stage percentages. Likewise, Bianchi et al. (50) did not observe significant differences in sleep efficiency between individuals with mild and severe OSAS, while the same authors reported significant differences in percentages of REM, N2 and N3, but not N1 between individuals with mild and severe OSAS. In addition, Andreou et al. (51) reported that N1, N3 and REM decreased, and N2 increased in individuals with OSAS when compared to a control group.

Next, similar to present study, Ratnavadivel et al. (52) reported more N1 and less deep sleep in individuals with OSAS than in healthy controls. Furthermore, Ng and Guan (53) reported that individuals with severe OSAS spent more time in REM-sleep than in deep or light sleep.

We also observed that participants with severe OSAS had a significantly higher arousal index than their counterparts with no or with mild or moderate OSAS.

As regards the wake index, this was higher in participants with moderate and severe OSAS than in participants with no or with moderate OSAS. This confirms previous findings (33, 50, 54).

For sleep spindles, these are considered as markers of N2 or nREM sleep microstructures (55, 56). Previous studies have investigated this neurophysiologic phenomenon either only in N2 (22, 37, 40, 57, 58) or in both N2 and N3 stages (58). In the present study, we performed spindle analyses separately for N2 and N3. More specifically, we investigated sleep spindle density and duration in N2 in frontal, central and occipital sites. In addition, for the first time, the duration and density of spindles were investigated separately in N3 stage. Although spindle density did gradually decrease with increasing OSA severity (that is, from no OSA to mild, moderate and severe OSA) the difference between groups was significant in N3, but not in N2. Thus, it appears that, for the first time, differences in sleep spindle signatures were observed in N3 between individuals without and with different degrees of OSAS. Interestingly, a dichotomy in spindle density was found: participants with no or with mild OSAS contrasted clearly with those participants with moderate and severe OSA. Based on this result, we suggest that apnea-hypopnea events below the threshold of 15 events/h may not disrupt normal sleep spindle activity. Or to put it the other way around: While mild OSA does not appear to disrupt the neurophysiological course of spindle density, moderate to severe OSAS does have a negative impact on spindle density.

Next, in N3, spindle density gradually decreased from frontal to occipital electrodes among all participants, and group differences also declined from the frontal to occipital area. To put it another way: Spindle density differences between the four groups were more pronounced in the frontal area than in the occipital area. To summarize, we believe that the progressive decline in spindle density with increasing OSAS severity reflects its disruptive effect on the synchronization of the spindle pattern generator in N3 stage.

As in the present study, Schonwald et al. (22) found no significant differences in spindle density between individuals with mild or moderate OSAS and healthy controls in the N2 stage. However, the present study expands upon their results as we investigated spindle density indices in both N2 and N3. Himannen et al. (37) found no significant differences between individuals with and without OSAS in visually detected bi-hemispheric synchronous spindle density during NREM sleep. However, they collapsed N2 and N3 in their analysis while in the present study we analyzed N2 and N3 separately.

Similarly, Huupponen et al. (59) reported no significant differences between individuals with and without OSAS in the total number of spindles. These authors employed an automatic spindle detector and analyzed the whole night sleep-EEG regardless of sleep stages. These differences might explain why the present results did not match those of Huupponen et al. (59).

Ondze et al. (33) found a lower spindle density during NREM sleep (both N2 and N3) in individuals with mild SDB than in healthy controls, but they found no significant differences between individuals with and without OSAS in REM spindle density.

Interestingly, Madaeva et al. (40) reported a higher number of spindles and higher spindle density during N2 in overweight adolescents with OSAS than in either overweight or normal weight healthy controls. As in the present study, Madaeva et al. (40) used an automatic software method to detect sleep spindles. As mentioned, previous studies of adults have investigated spindle density either solely in N2 (40), or for the whole sleep (59), or in NREM sleep (33), while no other study has, as in the present case, focused specifically on N3. One study of children examined spindle density separately in N2 and N3 (39) but its findings are the reverse of our own.

As regards spindle duration, this differed significantly between groups in N2 (except for C3 and O2 electrodes) but not in N3. We also note that only a few studies have investigated spindle duration in individuals with OSAS. In contrast to the present results, Schonwald et al. (22) did not observe significant differences in the spindle duration in N2 when comparing individuals with mild and moderate OSAS to healthy controls. Similarly, Madaeva et al. (40) reported no significant differences in spindle duration during N2 between overweight adolescent individuals with OSAS and either overweight or normal weight healthy controls.

Next, we also found significant associations between a higher spindle density and a lower AHI in N3, and between a longer spindle duration and a lower AHI in N2. Similarly, Li et al. (36) reported a significant association between a higher number of sleep spindle in N3 and a lower AHI, independently of sleep efficiency. Likewise, Madaeva et al. (40) reported significant associations between more favorable spindle characteristics and a lower AHI, a higher SaO2, and a lower total arousal index.

To summarize, our results show that OSAS produces a significant disruption of spindle density in N3, but not in N2. Unlike density, spindle duration was lower in moderate/severe OSAS during N2. It seems that, in individuals with moderate and severe OSAS, first the duration of spindles decreases during N2, and then the numbers of the spindles decrease in N3. The gradual decline in spindle duration in N2 may have led to reduction to such a level as to be too short to identify spindle activities. A similar process might have occurred for spindle density decline in N3. Our results also revealed that in contrast to moderate and severe OSAS, the mild condition of the disorder does not have a significant adverse effect on sleep spindle characteristics.

Some researchers have excluded apnea and hypopnea events so as to minimize any potential confounding effect caused by alpha activity in the automatic detection of slow spindles (22, 57). But respiratory events such as apnea have been shown to affect EEG frequency (29, 60). Dingli et al. (29) reported a significant elevation in sigma power among individuals with OSAS following respiratory events associated with an arousal (vs. without an arousal) during NREM and total sleep. Although they linked this finding to arousal-induced EMG activity and its effect on EEG, increased sigma power might reflect the hypothesized sleep maintenance function of spindle activity (34). Apnea and hypopnea events were not filtered out from the analysis in the present study because of the critical impact of these events on EEG activity.

Overall, the present controversy in the scientific literature as regards the occurrence (or otherwise) of sleep spindles in individuals with OSAS may be due to methodological issues, the use of different methodologies for spindle detection, choice of different sleep stages for analysis, and investigation of different characteristics such as sigma band power, total number of spindles, spindle density and duration.

Despite the novelty of the results, the following limitations should be taken into consideration. First, while we divided the OSA sample into subgroups on the basis of OSA severity, the resulting small sample sizes might have precluded more fine-grained patterns of results. However, the statistical analyses were also based on effect size calculations, which by definition are not sensitive to sample sizes. Second, we made a whole night EEG analysis, and dynamic changes in spindles and densities across the night may have influenced the results. Or simply put, no time-of-night effects were tested, although these were found to be relevant in earlier reports. Third, we didn't perform any analysis on fast vs. slow spindle characteristics. Fourth, we used a relatively simple and efficient spindle detection method providing a good trade-off between performance and computational complexity with few adjustable parameters. There are, however, more efficient tools with higher complexities, which require parameter optimization using multi-objective evolutionary algorithms as suggested in Chokroverty et al. (48). Next, the temporal evolution of slow and fast spindles in terms of amplitude and frequency and their density over the course of a full night's sleep can provide valuable information about the influence of OSA severity on spindle frequency distribution. These limitations highlight an important focus for future investigations. In addition, no control for multiple comparisons was considered. Further, there is evidence that a shortened sleep spindle duration was associated with dementia (28). In this line, a lower sleep spindle activity was associated with a lower cognitive performance (11–14, 21–24), and social competences in primary school children (25–27). It follows that future studies on sleep spindle indices in individuals with OSA should assess also participants' cognitive processes. Next, longitudinal studies might investigate, if among individuals with OSA sleep spindle indices are related to a lower quality of life (5), an increased risk of systemic comorbidities such as cardiovascular diseases, hypertension, and metabolic syndrome (6–8). Last, the quality of the data did not allow to test the hypothesis, if group differences in N3 sleep, but not so much in N2 sleep, was the result of an increasing frequency of apnea events during the course of the deepening of sleep.

## Conclusion

Although sleep spindles are primarily considered as characteristics of the N2 stage, our results showed that an increasing OSA severity was associated with a significant disruption of spindle density in N3, but not in N2. We recommend investigation of spindle characteristics in both N2 and N3 in future studies. Unlike density, spindle duration was lower in individuals with moderate and severe OSAS during the N2 stage. Our results also revealed that, in contrast to moderate and severe OSAS, the mild condition of the disorder does not have a significant adverse effect on sleep spindle characteristics.

## Data Availability Statement

The raw data supporting the conclusions of this article will be made available by the authors, without undue reservation.

## Ethics Statement

The studies involving human participants were reviewed and approved by Kermanshah University of Medical Sciences, Kermanshah, Iran. The patients/participants provided their written informed consent to participate in this study.

## Author Contributions

All authors listed have made a substantial, direct and intellectual contribution to the work, and approved it for publication.

## Conflict of Interest

The authors declare that the research was conducted in the absence of any commercial or financial relationships that could be construed as a potential conflict of interest.
