# Supplementary material for: Sleep Spindle Characteristics in Obstructive Sleep Apnea Syndrome (OSAS)
Source: Front Neurol. 2021 Feb 25;12:598632. doi: 10.3389/fneur.2021.598632 (PMC7947924; doi:10.3389/fneur.2021.598632)
Supplement: Supplementary file 1 [file Data_Sheet_1.docx]

Table S1. Median and interquartile range of spindle density (/min) for patients with OSA and normal sleepers in N2 in different measurement sites.

| Channel | Group | First quartile | Median | Third quartile | Interquartile Range |
| --- | --- | --- | --- | --- | --- |
| C3 | Control | 0.9197 | 1.1333 | 1.5521 | 0.6324 |
|  | Mild | 0.5892 | 1.2993 | 2.1157 | 1.5266 |
|  | Moderate | 0.5930 | 0.9134 | 1.4125 | 0.8195 |
|  | Severe | 0.5318 | 0.9061 | 1.8478 | 1.3160 |
| C4 | Control | 0.9144 | 1.1438 | 1.4390 | 0.5246 |
|  | Mild | 0.5662 | 1.3447 | 2.0657 | 1.4994 |
|  | Moderate | 0.8643 | 1.0400 | 1.5053 | 0.6410 |
|  | Severe | 0.5428 | 0.7808 | 1.5640 | 1.0212 |
| F3 | Control | 0.9375 | 1.1595 | 1.6538 | 0.7163 |
|  | Mild | 0.5532 | 1.2810 | 1.6945 | 1.1412 |
|  | Moderate | 0.5020 | 0.9562 | 1.4197 | 0.9178 |
|  | Severe | 0.5094 | 0.9102 | 1.7735 | 1.2641 |
| F4 | Control | 0.9322 | 1.1083 | 1.6125 | 0.6803 |
|  | Mild | 0.5121 | 1.1104 | 1.6890 | 1.1769 |
|  | Moderate | 0.7922 | 1.0532 | 1.5878 | 0.7956 |
|  | Severe | 0.6090 | 0.7237 | 1.6660 | 1.0571 |
| O1 | Control | 0.6284 | 0.8604 | 1.4625 | 0.8341 |
|  | Mild | 0.4281 | 1.0081 | 1.2909 | 0.8629 |
|  | Moderate | 0.4112 | 0.8288 | 1.5336 | 1.1224 |
|  | Severe | 0.3900 | 0.5652 | 1.4985 | 1.1085 |
| O2 | Control | 0.6388 | 0.9437 | 1.3000 | 0.6612 |
|  | Mild | 0.4318 | 0.9866 | 1.5099 | 1.0781 |
|  | Moderate | 0.6670 | 0.9482 | 1.2383 | 0.5713 |
|  | Severe | 0.4370 | 0.6048 | 1.3974 | 0.9603 |

Table S2. Median and interquartile range of spindle density (/min) for patients with OSA and normal sleepers in N3 in different measurement sites.

| Channel | Group | First quartile | Median | Third quartile | Interquartile Range |
| --- | --- | --- | --- | --- | --- |
| C3 | Control | 0.7278 | 1.4765 | 2.1293 | 1.4015 |
|  | Mild | 0.7214 | 1.2184 | 1.9019 | 1.1804 |
|  | Moderate | 0.0302 | 0.1919 | 0.7324 | 0.7022 |
|  | Severe | 0.0521 | 0.2022 | 0.9603 | 0.9082 |
| C4 | Control | 0.7612 | 1.4771 | 2.3983 | 1.6371 |
|  | Mild | 0.5530 | 1.0973 | 1.8414 | 1.2885 |
|  | Moderate | 0.0301 | 0.1857 | 0.6656 | 0.6355 |
|  | Severe | 0.0480 | 0.3403 | 0.9457 | 0.8978 |
| F3 | Control | 0.8396 | 1.6163 | 2.5965 | 1.7569 |
|  | Mild | 0.6708 | 1.2320 | 2.0523 | 1.3815 |
|  | Moderate | 0.0550 | 0.2346 | 0.6761 | 0.6210 |
|  | Severe | 0.0709 | 0.2129 | 0.7724 | 0.7015 |
| F4 | Control | 0.8250 | 1.6225 | 2.3958 | 1.5708 |
|  | Mild | 0.5650 | 1.1903 | 1.8864 | 1.3214 |
|  | Moderate | 0.0374 | 0.2336 | 0.6458 | 0.6084 |
|  | Severe | 0.0688 | 0.3424 | 0.7516 | 0.6827 |
| O1 | Control | 0.5771 | 1.0949 | 2.1064 | 1.5293 |
|  | Mild | 0.4855 | 0.7365 | 1.6346 | 1.1491 |
|  | Moderate | 0.0312 | 0.1137 | 0.5311 | 0.4999 |
|  | Severe | 0.0667 | 0.2234 | 0.7537 | 0.6869 |
| O2 | Control | 0.5875 | 1.0313 | 1.9544 | 1.3669 |
|  | Mild | 0.4627 | 0.7790 | 1.4814 | 1.0187 |
|  | Moderate | 0.0197 | 0.0772 | 0.4654 | 0.4456 |
|  | Severe | 0.0459 | 0.2265 | 0.6096 | 0.5637 |

Table S3. Median and interquartile range of spindle duration (in sec) for patients with OSA and normal sleepers in N2 in different measurement sites.

| Channel | Group | First quartile | Median | Third quartile | Interquartile Range |
| --- | --- | --- | --- | --- | --- |
| C3 | Control | 0.9228 | 0.9503 | 1.1968 | 0.2741 |
|  | Mild | 0.8490 | 1.0004 | 1.0504 | 0.2014 |
|  | Moderate | 0.7479 | 0.7973 | 0.9986 | 0.2507 |
|  | Severe | 0.7872 | 0.8355 | 0.9622 | 0.1751 |
| C4 | Control | 0.9007 | 0.9576 | 1.1876 | 0.2870 |
|  | Mild | 0.8349 | 0.8776 | 1.0171 | 0.1822 |
|  | Moderate | 0.7963 | 0.8292 | 1.1173 | 0.3211 |
|  | Severe | 0.7448 | 0.8224 | 0.9266 | 0.1818 |
| F3 | Control | 0.9757 | 1.0383 | 1.1356 | 0.1600 |
|  | Mild | 0.8468 | 0.8887 | 0.9487 | 0.1019 |
|  | Moderate | 0.7955 | 0.8253 | 0.8592 | 0.0637 |
|  | Severe | 0.8275 | 0.8660 | 1.0126 | 0.1851 |
| F4 | Control | 0.9678 | 1.0107 | 1.1254 | 0.1576 |
|  | Mild | 0.8389 | 0.8711 | 0.9811 | 0.1422 |
|  | Moderate | 0.8092 | 0.8499 | 0.9147 | 0.1054 |
|  | Severe | 0.7485 | 0.8657 | 0.9691 | 0.2206 |
| O1 | Control | 0.7754 | 0.9109 | 1.0211 | 0.2457 |
|  | Mild | 0.7683 | 0.8212 | 0.8752 | 0.1068 |
|  | Moderate | 0.7200 | 0.7746 | 0.8515 | 0.1315 |
|  | Severe | 0.7173 | 0.7930 | 0.8360 | 0.1187 |
| O2 | Control | 0.7959 | 0.8675 | 0.9870 | 0.1911 |
|  | Mild | 0.7877 | 0.8672 | 0.9093 | 0.1216 |
|  | Moderate | 0.7197 | 0.7852 | 0.9091 | 0.1894 |
|  | Severe | 0.7440 | 0.7807 | 0.8343 | 0.0902 |

Table S4. Median and interquartile range of spindle duration (in sec) for patients with OSA and normal sleepers in N3 in different measurement sites.

| Channel | Group | First quartile | Median | Third quartile | Interquartile Range |
| --- | --- | --- | --- | --- | --- |
| C3 | Control | 0.8597 | 0.9133 | 1.0967 | 0.2370 |
|  | Mild | 0.7634 | 0.8375 | 0.9708 | 0.2074 |
|  | Moderate | 0.7499 | 0.8021 | 1.3320 | 0.5821 |
|  | Severe | 0.7443 | 0.7749 | 0.9945 | 0.2501 |
| C4 | Control | 0.8260 | 0.8959 | 1.1093 | 0.2834 |
|  | Mild | 0.7623 | 0.8219 | 0.9326 | 0.1703 |
|  | Moderate | 0.7539 | 0.7878 | 1.0322 | 0.2782 |
|  | Severe | 0.7228 | 0.7883 | 0.9124 | 0.1896 |
| F3 | Control | 0.8590 | 0.9958 | 1.1514 | 0.2924 |
|  | Mild | 0.8091 | 0.8264 | 0.8886 | 0.0796 |
|  | Moderate | 0.7682 | 0.8374 | 0.9187 | 0.1505 |
|  | Severe | 0.7846 | 0.8260 | 1.0504 | 0.2658 |
| F4 | Control | 0.8718 | 0.9339 | 1.0309 | 0.1591 |
|  | Mild | 0.7464 | 0.7986 | 0.8823 | 0.1359 |
|  | Moderate | 0.7472 | 0.8424 | 0.8993 | 0.1521 |
|  | Severe | 0.7567 | 0.8617 | 0.9539 | 0.1971 |
| O1 | Control | 0.7733 | 0.8225 | 0.9047 | 0.1314 |
|  | Mild | 0.7118 | 0.7425 | 0.8063 | 0.0944 |
|  | Moderate | 0.7088 | 0.7852 | 0.8866 | 0.1778 |
|  | Severe | 0.6824 | 0.7766 | 0.9072 | 0.2249 |
| O2 | Control | 0.7426 | 0.8077 | 0.8973 | 0.1547 |
|  | Mild | 0.7148 | 0.7583 | 0.8265 | 0.1117 |
|  | Moderate | 0.6916 | 0.7170 | 0.8204 | 0.1288 |
|  | Severe | 0.6832 | 0.7331 | 0.8033 | 0.1201 |
